# Removing celiac disease-related gluten proteins from bread wheat while retaining technological properties: a study with Chinese Spring deletion lines

**DOI:** 10.1186/1471-2229-9-41

**Published:** 2009-04-07

**Authors:** Hetty C van den Broeck, Teun WJM van Herpen, Cees Schuit, Elma MJ Salentijn, Liesbeth Dekking, Dirk Bosch, Rob J Hamer, Marinus JM Smulders, Ludovicus JWJ Gilissen, Ingrid M van der Meer

**Affiliations:** 1 Plant Research International, Wageningen UR, PO Box 16, NL-6700 AA Wageningen, The Netherlands; 2 Laboratory of Food Chemistry, Wageningen UR, PO Box 8129, NL-6700 EV Wageningen, The Netherlands; 3 Allergy Consortium Wageningen, PO Box 16, NL-6700 AA Wageningen, The Netherlands; 4 Leiden University Medical Center, PO Box 9600, NL-2300 RC Leiden, The Netherlands; 5 Dynomics BV, Erasmus Medical Centre, Department of Immunology, PO Box 82, NL-1400 AB Bussum, The Netherlands

## Abstract

**Background:**

Gluten proteins can induce celiac disease (CD) in genetically susceptible individuals. In CD patients gluten-derived peptides are presented to the immune system, which leads to a CD4^+ ^T-cell mediated immune response and inflammation of the small intestine. However, not all gluten proteins contain T-cell stimulatory epitopes. Gluten proteins are encoded by multigene loci present on chromosomes 1 and 6 of the three different genomes of hexaploid bread wheat (*Triticum aestivum*) (AABBDD).

**Results:**

The effects of deleting individual gluten loci on both the level of T-cell stimulatory epitopes in the gluten proteome and the technological properties of the flour were analyzed using a set of deletion lines of *Triticum aestivum *cv. Chinese Spring. The reduction of T-cell stimulatory epitopes was analyzed using monoclonal antibodies that recognize T-cell epitopes present in gluten proteins. The deletion lines were technologically tested with respect to dough mixing properties and dough rheology. The results show that removing the α-gliadin locus from the short arm of chromosome 6 of the D-genome (6DS) resulted in a significant decrease in the presence of T-cell stimulatory epitopes but also in a significant loss of technological properties. However, removing the ω-gliadin, γ-gliadin, and LMW-GS loci from the short arm of chromosome 1 of the D-genome (1DS) removed T-cell stimulatory epitopes from the proteome while maintaining technological properties.

**Conclusion:**

The consequences of these data are discussed with regard to reducing the load of T-cell stimulatory epitopes in wheat, and to contributing to the design of CD-safe wheat varieties.

## Background

Celiac disease (CD) is a disorder that is characterized by a permanent intolerance to gluten proteins from wheat, rye, and barley. Over 0.5% of the Western population suffers from CD, which presents itself by chronic diarrhea, fatigue, osteoporosis, lymphoma, and several other clinical symptoms after prolonged gluten consumption. Until now, the only treatment is a complete and life long elimination of gluten from the daily diet [1]. In the small intestine, several native gluten peptides can bind directly to specific human leukocyte antigen (HLA)-DQ2 or DQ8 receptors on antigen presenting cells (APCs). However, after deamidation by tissue transglutaminase (tTG), the affinity of the peptides for these HLA-receptors is strongly increased. The gluten peptides can be presented by APCs to gluten-sensitive T-cell lymphocytes leading to the release of cytokines, which will cause inflammation reactions and result in damaged intestinal villi [2].

Gluten are major storage proteins and have many interesting characteristics for food industrial applications, e.g. in baking bread. Gluten proteins can be divided into three main groups: high molecular weight glutenin subunits (HMW-GS), low molecular weight glutenin subunits (LMW-GS), and gliadins. The HMW-GS are divided in x-type and y-type subunits [3]. The LMW-GS are divided into B-, C-, and D-type subunits [4]. Gliadins are divided into α/β-, γ-, and ω-gliadins [5]. Multiple T-cell activating gluten peptides were mainly found in α-gliadins, but also in γ-gliadins and both LMW-GS and HMW-GS [1,2,6,7]. Especially peptides derived from α-gliadins are recognized by T-cells from most CD patients, while T-cell responses to γ-gliadins and glutenins are less frequently found [2,7-10].

Wheat varieties with very low amounts of T-cell stimulatory epitopes may be tolerated by many CD-patients [9,11], while a diet based on wheat varieties reduced in T-cell stimulatory epitopes may help in the prevention of CD, as it has been observed that the amount and duration to gluten exposure is associated with the initiation of CD [12-14]. Breeding for bread wheat (*Triticum aestivum*) with less T-cell stimulatory gluten may result, however, in varieties with unwanted loss of technological properties, because glutenins and gliadins together contribute largely to dough quality. A correct mixture of both glutenins and gliadins is essential to obtain optimal viscoelastic dough [15], and the quantity and the size distribution of the gluten proteins are important factors for polymerization [16,17].

Gluten-encoding genes are located on the three homoeologous genomes of bread wheat: A, B, and D. A few (for HMW-GS) to a hundred (for α-gliadins) gene copies are present in hexaploid wheat. Sequences of individual gene copies within the same gluten family, such as the α-gliadins, are very similar and may contain multiple and different T-cell stimulatory epitopes [18]. Gluten proteins are encoded by 15 major loci. The HMW-GS are encoded by loci on the long arm of group 1 chromosomes (*Glu-A1, -B1*, and -*D1*) [19]. The LMW-GS are mainly encoded by the *Glu-3 *loci on the short arms of group 1 chromosomes (*Glu-A3*, -*B3*, and -*D3*) [20] and are tightly linked to the loci encoding the γ-gliadins and ω-gliadins (*Gli-A1,-B1*, and -*D1 *and *Gli-A3*, -*B3*, and -*D3*). Most α/β-gliadins are encoded by loci on the short arms of group 6 chromosomes (*Gli-A2*, *B2*, and *D2*) [21].

In this study, deletion lines of *Triticum aestivum *cv. Chinese Spring (CS) were selected [22-24]. These deletion lines are generally lacking one locus containing gluten genes from one of the three homoeologous chromosomes. Here, we explore the feasibility to reduce T-cell stimulatory epitopes in hexaploid bread wheat by screening with epitope-specific monoclonal antibodies [25-27], while maintaining the technological properties.

## Results

### Protein database search

The NCBI protein database search was performed to analyze the number of proteins that contain the different sequences recognized by mAb and T-cells. This search provided insight in how many proteins were expected to contain the different sequences and which different sequences were present within the proteins. The numbers of protein sequences that contain the various sequences involved in the onset of CD that are recognized by T-cells and mAbs are shown in Table 1. It was observed that the mAb and T-cell minimal sequences were specific for the epitopes in each of the expected protein group, with the exception of the mAb recognizing Glia-α9, whose minimally recognized sequence was also present in a number of γ- and ω-gliadin proteins. The sequence recognized by the T-cells was not present within any other protein group except for the α/β-gliadins. The minimal sequences recognized by mAbs LMW-1 and LMW-2 were more frequently found in the LMW-GS group than the sequence recognized by the corresponding T-cells. The sequences recognized by mAb and T-cells for HMW-glt was present in nearly all HMW-GS protein sequences.

**Table 1:** Results of database search for sequences recognized by mAbs and T-cells.

	Protein groups				
	
Epitope	α/β-gliadins	γ-gliadins	ω-gliadins/D-type LMW-GS	LMW-GS	HMW-GS
mAb Glia-α9 (QPFPQPQ)	68	67	3	-	-
T-cell Glia-α9 (PFPQPQLPY)	44	-	-	-	-

mAb Glia-α20 (RPQQPYP)	48	-	-	-	-
T-cell Glia-α20 (FRPQQPYPQ)	48	-	-	-	-

mAb LMW-1 (PPFSQQ)	-	-	-	233	-
mAb LMW-2 (QSPF)	-	-	-	163	-
T-cell LMW-glt (PFSQQQQSPF)	-	-	-	21	-

mAb HMW-glt (QGQQGYYP)	-	-	-	-	67
T-cell HMW-glt (QGYYPTSPQ)	-	-	-	-	65

Number of sequences retrieved	84	93	6	263	67

The number in each cell represents the presence of the recognized sequence by mAb or T-cell within a protein group. '-' in a cell means that the sequence was not present.

### SDS-PAGE

**Figure 1:**
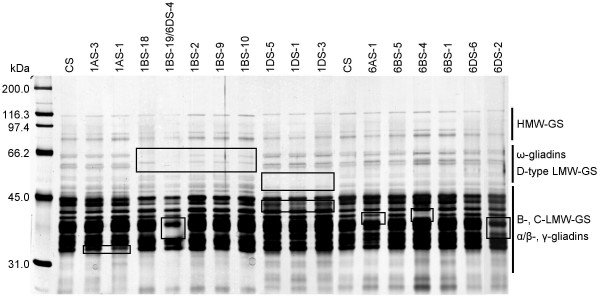
**SDS-PAGE analysis of prolamin extracts from Chinese Spring deletion lines**. CS: Chinese Spring wild type. Boxes indicate differences in protein bands.

**Figure 2:**
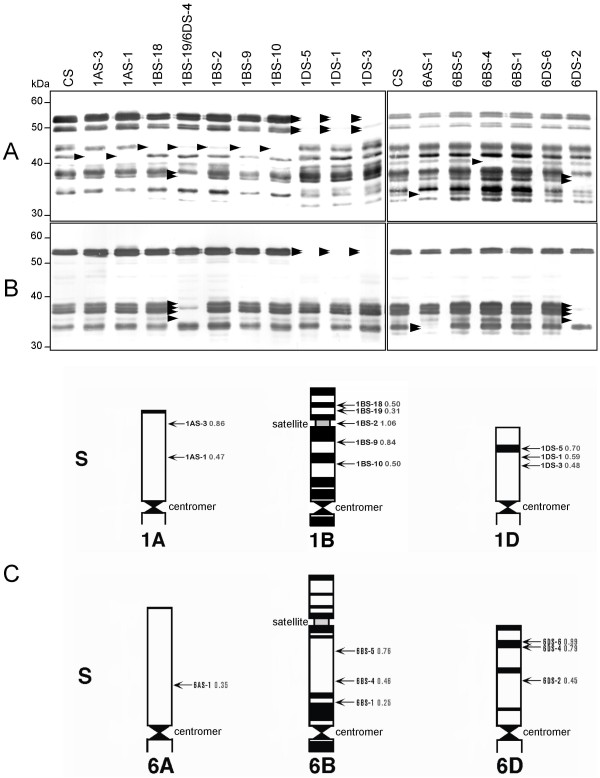
**Immunoblot analysis of Chinese Spring deletion lines of the short arm of chromosome 1 and 6**. (A) Using mAb Glia-α9. (B) Using mAb Glia-α20. CS: Chinese Spring wild type. Arrowheads indicate absent protein bands. (C) Physical maps of the short (S) arms of wheat chromosomes 1A, 1B, 1D, 6A, 6B, and 6D from centromer to telomeric ends (Wheat Genetic and Genomic Resources Centre, Kansas State University, USA). Arrows on the right of each chromosome indicate the deletion lines with their breakpoint (indicated as fraction length from the centromer). The banding patterns within the chromosomes are according to Gill et al. [24]

To obtain the gluten protein patterns from the CS deletion lines, gluten proteins were extracted and analyzed by SDS-PAGE followed by silver staining. Major differences compared to CS wild type are indicated by boxes in Figure 1. Differences in gluten protein content compared to CS wild type were mostly observed in the B-, C- type LMW-GS and α/β-, γ-gliadin region. Lines with deletions of the short arms of chromosomes 1D were missing several gluten protein bands in the ω-gliadin/D-type LMW-GS region. The double deletion line, 1BS-19/6DS-4 (Figure 1), was missing the largest number of gluten protein bands because of two deletions in gluten encoding regions. Unexpected results were obtained for deletion line 1BS-18, which is the line with the smallest deletion of chromosome arm 1BS. This line is missing an extra band compared to the other 1BS deletion lines having larger deletions. This does not fit with reported results on deletion lines [22,23]. Deletion line 6BS-4 (Figure 1) missed a gluten protein band that is present in the other deletion lines of chromosome 6B, even though deletion line 6BS-1 has been reported (WGGRC; Figure 2C) to contain a larger deletion than 6BS-4. Deletion line 6BS-4 also contains the 5BS-2 deletion, but, to our knowledge, no gluten protein locus has ever been identified onto the short arm of chromosome 5B. We do not have any explanation for these discrepancies

### *Gli-1 *deletions

CS deletion lines were analyzed for their contribution to T-cell stimulatory epitopes by using various mAbs recognizing different T-cell epitopes. In Figure 2A, immunoblot results are presented using mAbs Glia-α9 and Glia-α20 for deletion lines of the short arm of chromosomes 1 (*Gli-1*) and 6 (*Gli-2*). Major differences, compared to CS wild type, are indicated with arrowheads. Deletion lines 1AS-3 and 1AS-1 were missing one gluten protein band by using mAb Glia-α9 and no gluten protein bands by using mAb Glia-α20 (Figures 2A and 2B). This suggests that this missing gluten protein only contains the epitope sequence recognized by mAb Glia-α9 and the loci encoding these gluten protein map to bin 1AS3-0.86–1.00 (the terminal 14% of chromosome arm 1AS) (Figure 2C). All five deletion lines of the short arm of chromosome 1B (Figure 2A) lacked one gluten protein band by using mAb Glia-α9 and no gluten protein band by using mAb Glia-α20. The double deletion line 1BS-19/6DS-4 (Figure 2A) was missing two extra bands using mAb Glia-α9 and four by using mAb Glia-α20, which is caused by the 6DS-4 deletion. Two gluten protein bands were recognized by both mAbs Glia-α9 and Glia-α20. All 1BS deletion lines (Figure 2A) lacked the same gluten protein band recognized by mAb Glia-α9 and because of that the loci encoding corresponding gluten protein map to bin 1BSsat18-0.50–1.00 (Figure 2C). All three deletion lines of the short arm of chromosome 1D (Figure 2A) lacked four gluten protein bands by using mAb Glia-α9 and two gluten protein bands by using mAb Glia-α20. These missing protein bands correspond to the boxed (missing) proteins in Figure 1. One gluten protein band did not completely disappear by using mAb Glia-α9. This is probably because of the presence of gluten proteins from different loci but having the same molecular weights, therefore becoming visible only as one gluten protein band. The loci encoding the recognized gluten proteins map to bin 1DS5-0.7–1.0 (the terminal 30% of 1DS) (Figure 2C). The two gluten protein bands recognized by mAb Glia-α20 were the same as recognized by mAb Glia-α9.

### *Gli-2 *deletions

When analyzing CS deletion lines that are lacking parts of the short arm of chromosome 6, deletion line 6AS-1 (Figure 2A) lacked one gluten protein band in immunoblotting using mAb Glia-α9 and two bands by using mAb Glia-α20. Deletion line 6BS-4 (Figure 2A) lacked one gluten protein band by using mAb Glia-α9, but this was not the case for the other two 6BS deletion lines, 6BS-1 and 6BS-5 (Figure 2A), which is not consistent with the reported sizes of the deletions. In the 6BS deletion lines, no changes were observed in gluten protein bands compared with CS wild type by using mAb Glia-α20 (Figure 2B). These results suggest that the short arm of chromosome 6B encodes no gluten proteins containing T-cell stimulatory epitopes recognized by both mAbs Glia-α9 and Glia-α20, at least not mapping to bin 6BS-0.25–1.00 (terminal 75% of 6BS) (Figure 2C). Deletion line 6DS-2, the line with the largest deletion (Figures 2A and 2B) lacked two gluten protein bands recognized by mAb Glia-α9 and four bands by mAb Glia-α20. One gluten protein band has not completely disappeared probably because of the presence of different gluten proteins having the same molecular weight within one gluten protein band. The same gluten protein bands are also absent in the double deletion line 1BS-19/6DS-4 (Figure 2A). These missing protein bands correspond to the boxed (missing) proteins in Figure 1. Hence, the loci encoding these gluten proteins map to bin 6DS4-0.79–0.99 (Figure 2C).

### *Glu-3 *deletions

**Figure 3:**
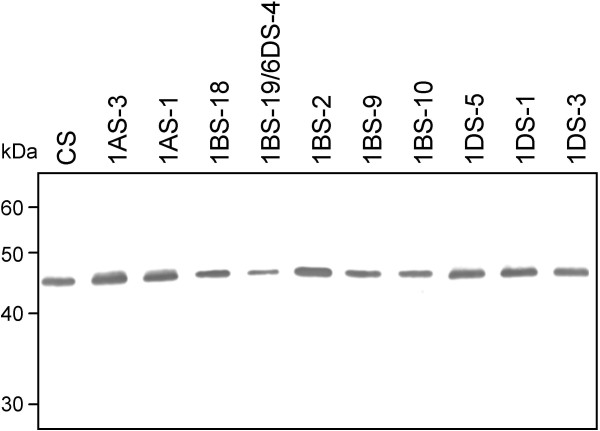
**Immunoblot analysis of Chinese Spring deletion lines of the short arm of chromosome 1, using mAb LMW-2**. CS: Chinese Spring wild type.

The immunoblot results using mAb LMW-2 for the deletion lines of the short arm of chromosome 1 are shown in Figure 3. One band was observed in all the deletion lines and in CS wild type without significant differences. Immunoblot results using mAb LMW-1 showed similar patterns (results not shown).

### *Glu-1 *deletions

**Figure 4:**
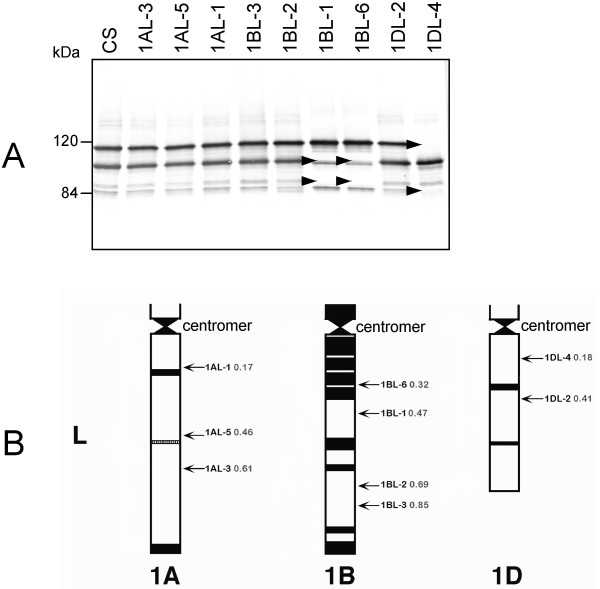
**Immunoblot analysis of Chinese Spring deletion lines of the long arm of chromosome 1**. (A) Using mAb HMW-glt. CS: Chinese Spring wild type. (B) Physical maps of the long (L) arms of wheat chromosomes 1A, 1B, and 1D from centromer to telomeric ends (Wheat Genetic and Genomic Resources Centre, Kansas State University, US). Arrows on the right of each chromosome indicate the deletion lines with their breakpoint (indicated as fraction length from the centromer). The banding patterns within the chromosomes are according to Gill et al [24].

Within the protein database, nearly all HMW-GS had epitope sequences recognized by mAb HMW-glt. The immunoblot results for the deletion lines of the long arm of chromosome 1 using the mAb recognizing HMW-glt are shown in Figure 4A. In CS wild type, all four HMW glutenin subunits were detected. No contribution to HMW-GS was observed for the long arm of chromosome 1A, as expected for a transcriptional silent locus. Two HMW-GS, 1Bx7 and 1By8, were absent in deletion lines 1BL-1 and 1BL-6. This suggests that the locus encoding HMW-GS 1Bx7 and 1By8 map to bin 1BL1-0.47–0.69 (Figure 4B). The two HMW-GS, 1Dx2 and 1Dy12, were absent in deletion line 1DL-4. This suggests that the loci encoding HMW-GS 1Dx2 and 1Dy12 map to bin 1DL4-0.18–0.41 (Figure 4B).

### Rheological parameters of Chinese Spring deletion lines

**Figure 5:**
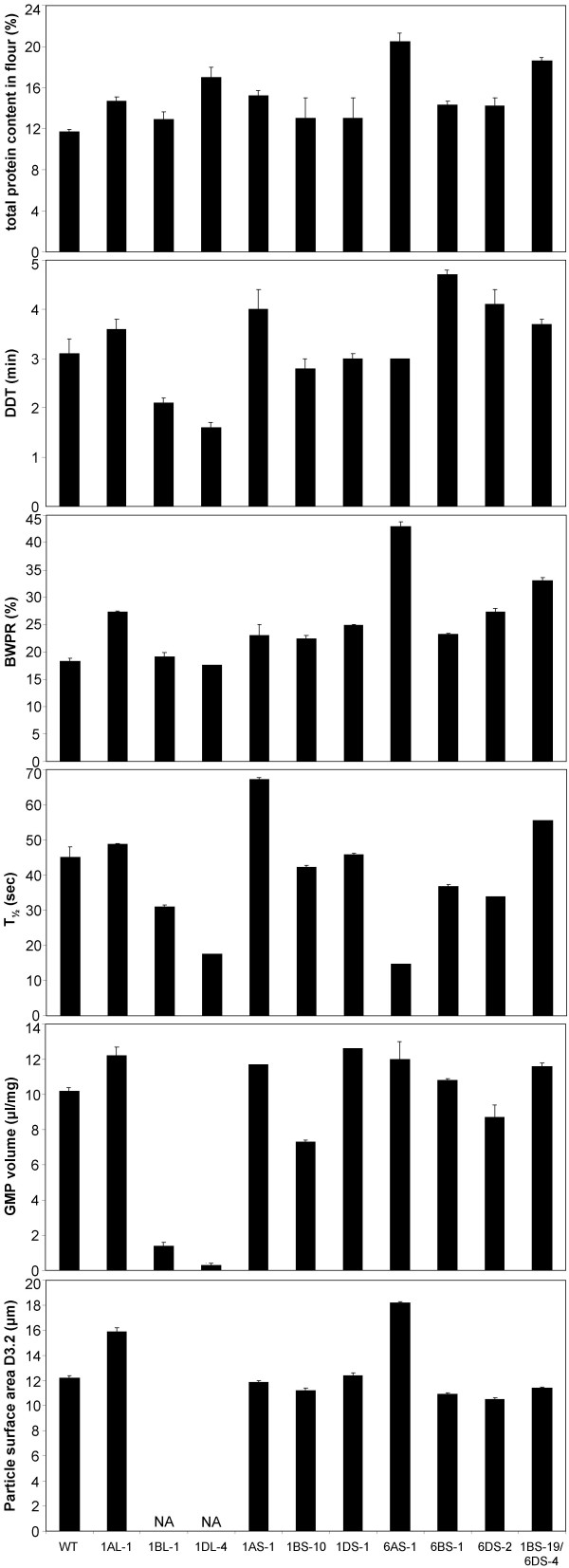
**Rheological parameters tested for Chinese Spring deletion lines**. All technological measurements were performed in duplicate, except the relaxation test (T_1/2_) for deletion lines 1DL-4, 6AS-1, 6DS-2, and 6DS-4/1BS-19. Error bars represent the standard error. 'NA' means not analyzed for particle surface area (D_3,2_) because the amount of GMP was too low.

The lines with the largest deletions from chromosomes 1 and 6, according to our results, were used for technological testing. Parameters among flours of different deletion lines are presented in Figure 5 and in the Additional file 1: Rheological parameters.

Total protein content in flour (% w/w) of all deletion lines was higher compared to CS wild type flour. Especially protein content in flour of line 6AS-1 was high (20.5%), followed by protein content in flour of deletion line 1BS-19/6DS-4 (18.6%).

The glutenin macro polymer (GMP) content expressed as volume per mg protein was decreased in deletion line 1BL-1 and was nil in deletion line 1DL-4 (Figure 5 and Additional file 1: Rheological parameters). GMP represents the highly aggregated glutenin protein network that is the prime determinant of dough elastic properties. A decrease in GMP is therefore expected to lead to a decrease in dough strength [28-30]. Because of the low amount of GMP present in flour of the deletion lines 1BL-1 and 1DL-4, it was impossible to estimate glutenin particle sizes for these lines. Flours of the two deletion lines, 1BS-10 and 6DS-2, showed a small decrease in GMP volume. For all other deletion lines, the GMP volume was increased.

Glutenin particle size is a predictor of dough mixing properties [31]. Average glutenin particle size was increased in flours of deletion line 1AL-1 and 6AS-1. In deletion lines 6DS-2, 6BS-1, 1BS-10, 1BS-19/6DS-4 and 1AS-1 the average particle size was decreased compared to CS wild type.

Dough made from flours of the two deletion lines 1BL-1 and 1DL-4, lacking HMW-GS, showed a significant decrease in dough development time (DDT) (Figure 5 and Additional file 1: Rheological parameters). Dough made from all other deletion lines showed increase in DDT, especially the lines with deletions of the short arm of chromosome 6 (6AS-1, 1BS-19/6DS-4, 6DS-2, and 6BS-1) and 1AS-1. Deletions of the *Gli-2 *loci seem to have a substantial effect on increasing DDT.

Bandwidth at peak resistance (BWPR) is a measure of dough stability. The BWPR was slightly decreased for deletion line 1DL-4 and was increased for all other deletion lines compared to CS wild type dough (Figure 5 and Additional file 1: Rheological parameters). The BWPR was especially high for deletion lines 6AS-1 and 1BS-19/6DS-4. It is relevant to note that these are the same deletion lines having the highest protein content in flour.

Dough elasticity, indicated by relaxation half time (T_1/2_), was decreased in flours of deletion lines 1BL-1 and 1DL-4, which lack HMW-GS, and in deletion lines 6BS-1 and 6DS-2 (Figure 5 and Additional file 1: Rheological parameters). In contrast, deletion lines 1BS-19/6DS-4 and 1AS-1, showed an increase in T_1/2_, indicating more elastic dough [32,33].

**Figure 6:**
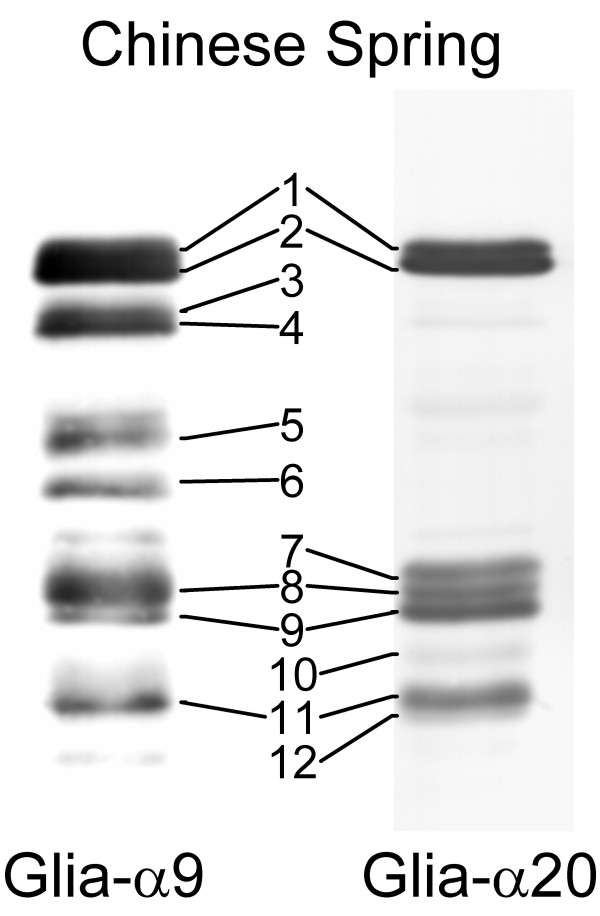
**Numbering of protein bands reacting with mAbs Glia-α9 and Glia-α20 in Chinese Spring wild type**. Immunoblots of Chinese Spring wild type using mAbs Glia-α9 (left) and Glia-α20 (right).

To summarize, in Figure 6 immunoblots are shown for Chinese Spring wild type and the gliadin proteins reacting with mAbs Glia-α9 and Glia-α20 are numbered. In Table 2 the relation is shown of these proteins together with their bin-location on the chromosomes and the rheological parameters if these proteins are missing in the deletion lines. Deletions of the long arms of chromosome 1A, 1B, and 1D are not included because the HMW-GS encoded by the loci on these arms (1BL and 1DL) seem to be required for good technological properties.

**Table 2:** Bin location of gliadin proteins and effect on rheological parameters if absent in deletion lines.

	mAb			Rheological parameters					
			
Gliadin protein bands in CS	Glia-α9	Glia-α20	Bin location	Total protein in flour (%)	DDT^a ^(min)	BWPR^b ^(%)	T_1/2_^c ^(sec)	GMP^d ^volume (μl/mg)	Particle surface area (D_3,2_) (μm)
1	yes	yes	1DS5-0.70–1.00	0	0	+	0	+	0
2	yes	yes	1DS5-0.70–1.00	0	0	+	0	+	0
3	yes	no	1DS5-0.70–1.00	0	0	+	0	+	0
4	yes	no	1DS5-0.70–1.00	0	0	+	0	+	0
5	yes	no	1BSsat0.50–1.00	0	0	+	0	-	-
6	yes	no	1AS3-0.86–1.00	+	+	+	+	+	0
7	no	yes	6DS4-0.79–0.99	+	+	+	-	-	-
8	yes	yes	6DS4-0.79–0.99	+	+	+	-	-	-
9	yes	yes	6DS4-0.79–0.99	+	+	+	-	-	-
10	yes	no	6DS4-0.79–0.99	+	+	+	-	-	-
11	yes	yes	6AS-0.35–1.00	+	0	++	--	+	++
12	no	yes	6AS-0.35–1.00	+	0	++	--	+	++

Chinese Spring wild type				0	0	0	0	0	0

Protein bands are numbered as shown in Figure 6 and their bin location is determined. Bin location is linked to rheological parameters. Results are compared to Chinese Spring, which is set at "0". "+" in cell means value is up to 50% higher. "++" in cell means value is between 50 to100% higher. "-" in cell means values is up to 50% lower. "--" in cell means value is between 50 to100% lower. "Yes" or "no" in cell means the reaction with the mAb.

^a^Dough development time

^b^Band Width at Peak Resistance

^c^Flow-relaxation half time

^d^Glutenin Macro Polymer

## Discussion

In this study, we examined the possibilities to develop a bread wheat variety with both reduced levels of T-cell stimulatory epitopes and good technological properties. We used a set of Chinese Spring deletion lines that lack different gluten protein-encoding loci from the group 1 and 6 chromosomes to determine whether reduction in T-cell stimulatory epitopes can be achieved by removal of certain gluten protein encoding genes with minimal effect on the technological properties of bread wheat. Many cytogenetic resources have been developed in *T. aestivum *cv Chinese Spring, which is considered as a model variety for hexaploid wheat. However, differences among varieties may exist.

### CD immunogenic epitopes

On the short arm of the group 6 chromosomes, the gluten loci that encode α-gliadins are located. The α-gliadins are considered the most immunogenic concerning both the adaptive immune response and the innate immune response [2,8,10,11]. We observed that the locus on the short arm of chromosome 6D, mapped to bin 6DS-0.45–0.99, is responsible for most of the T-cell stimulatory α-gliadin proteins. These results are in agreement with the results obtained by Molberg et al. [34] who showed no decrease in response of DQ2-α-II T-cells for deletion line 6DS-6 and a significant decrease in T-cell response for deletion lines 6DS-4 and 6DS-2. In addition, results are in agreement with results of Van Herpen et al. [18], based on relative presence of CD-epitopes in α-gliadin ESTs from the three homoeologous loci, and with results of Salentijn et al. [35] on the presence in cDNAs from two hexaploid and two tetraploid cultivars. When using mAb Glia-α20 in immunoblotting also two gluten protein bands were stained that were encoded by the short arm of chromosome 1D. We tentatively assign these as ω-gliadins/D-type LMW-GS containing the mAb Glia-α20 sequence. Only a few ω-gliadin proteins have been sequenced so far because they are difficult to clone due to the presence of large repetitive domains [36]. It has been shown that ω-gliadins may have epitopes that are involved in gluten-sensitive response of CD patients [37,38]. The α-gliadins encoded by chromosome 6 seem to be related to gliadins encoded by chromosome 1 from which they might have originated through gene duplication and/or translocation [39,40]. Analysis of the minimal sequence recognized by mAb Glia-α9 indicated that this sequence also occurs in some γ- and ω-gliadins. Indeed, mAb Glia-α9 recognized gluten protein bands that disappeared in deletion lines of the short arm of chromosome 1A, 1B, and 1D (where γ- and ω-gliadin encoding genes are located). We observed that genes mapped to bin 1DS-0.48–1.00 had the highest contribution to the number of T-cell stimulatory epitopes.

### Technological properties

Studies have shown that the technological parameters of wheat flours are influenced by alleles encoding different HMW-GS [41-44], LMW-GS [45,46], and gliadins [47].

Deleting parts of the short arm of chromosome 1A resulted in an increased dough development time (DDT) and volume of glutenin macro polymer (GMP). A decrease in LMW-GS or gliadins results in a relative increase of ratios for HMW-GS/LMW-GS or glutenins/gliadins. Such a change was suggested to increase dough strength [15,16]. Indeed, we found that removal of the locus from the short arm of chromosome 1A resulted in increased dough elasticity. In the deletion lines 1AS-1 and 1DS-1, higher GMP volumes were observed, while in deletion line 1BS-10 a decreased GMP volume was found together with decreased DDT. On chromosome 1B, also a *Glu-B2 *locus is located encoding a B-type LMW-GS [48,49] and a *Glu-B3 *locus is located encoding two tightly linked genes for an ω-gliadin and a B-type LMW-GS [50]. This suggests that LMW-GS encoded by these loci are important for the formation of the GMP [51,52]. Removal of the loci could affect the ratios for HMW-GS/LMW-GS or glutenins/gliadins. Chromosome 1D encodes a D-type LMW-GS containing a single cysteine residue and therefore may act as a chain terminator [53,54]. The absence of the protein could increase the GMP volume in deletion line 1DS-1. It would be expected that the GMP volume would decrease in deletion line 1AS-1 because of removal of the locus encoding major LMW-GS. We observed, however, that no T-cell stimulatory epitopes present in LMW-GS disappeared from the immunoblot using mAbs LMW-1 and LMW-2, which is possible if expression from the deleted locus is compensated for by the other two loci present on the homoeologous chromosomes, for example by a higher expression of *Glu-B3*. Compensation behavior of storage protein synthesis in wheat was observed by Wieser et al. [55] after inhibition of the expression of α-gliadins by RNA interference (RNAi). Also Gil-Humanes et al. [56] recently observed while RNAi reduced the proportion of γ-gliadins by 55–80% and α-gliadins by 63%, this did not lead to similar reduction in proteins detected by the sandwich ELISA using the R5 monoclonal antibody. The R5 assay was, however, developed for the detection of gluten proteins from different sources and not optimized to detect T-cell stimulatory gluten proteins [57]. Hence, although the R5 assay is currently considered the standard test for identification of gluten contaminants, we regarded this test unsuitable in the context of this study.

With respect to technological properties, deletion line 6AS-1 showed an increase in GMP volume and a strong increase in glutenin particle size. In contrast, deletion lines 6BS-1 and 6DS-2 showed a decrease in glutenin particle size and a decrease in GMP volume for deletion line 6DS-2. Gliadins of the α- and γ-type have been identified to contain an extra cysteine residue that makes them act as chain terminators. We suggest that the short arm of chromosome 6A in CS is encoding a chain terminating α-gliadin. The lower content of chain terminators could account for a larger size of glutenin particles as observed in deletion line 6AS-1. Because of compensation, deletions of the short arm of chromosome 6B and 6D could lead to an increased expression of chain terminating α-gliadins encoded by the short arm of chromosome 6A and result in observed smaller glutenin particle sizes. The deletions of the short arm of chromosome 6B and 6D resulted in stronger dough as shown by increased DDT. This effect on dough strength is expected because a decrease in α-gliadins results in a relative increase of the glutenin/gliadin ratio. The GMP volume of flour from deletion line 6DS-2 was decreased, which indicates weaker dough, whereas the DDT was increased, which indicates stronger dough. Because of this effect, the decrease in GMP volume in deletion line 6DS-2 resulted in decreased elasticity rather than decreased dough strength.

We observed that technological properties of flour from deletion lines were strongly affected by the removal of the different HMW-GS with the strongest effect in deletion line 1DL-4. Dough strength (as expressed as DDT and GMP volume) and dough elasticity (T_1/2_) were both strongly decreased, which is in agreement with published results [15,58,59]. Deletion of the locus on the long arm of chromosome 1A resulted in some increase in dough strength (DDT and GMP volume) and elasticity (T_1/2_). In addition, glutenin particle sizes were significantly increased. Both the x-type and y-type encoding genes of CS at *Glu-A1 *are silent [19]. In most studies, the silent locus at *Glu-A1 *was not found to be important to determine dough strength compared to non-silent loci [45,46], so the effect of deletion of the long arm of chromosome 1AL might be because of the absence of other gene products. Based on these results, the *Glu-1 *loci of CS are considered inappropriate as a focus to breed for wheat with less T-cell stimulatory epitopes if technological properties are to be preserved.

## Conclusion

A strategy to breed for bread wheat with less T-cell stimulatory gluten epitopes while retaining technological properties is feasible by focusing on eliminating genes present on the short arms of chromosome 1D and 6D. This will result in a wheat variety with highly decreased T-cell stimulatory epitopes. However, eliminating genes might decrease dough elasticity because of a changed ratio in glutenin and gliadin proteins. This ratio could be compensated for by the addition of monomeric proteins with no T-cell stimulatory to the flour, for example from safe sources like oats, or by the introduction through breeding or genetic modification of CD-safe gliadin genes. In addition, wheat varieties with limited but not complete reduced levels of T-cell stimulatory epitopes may still contribute to lower the gluten load for the entire population and it may reduce the development of CD in a number of potential patients.

## Methods

### Wheat materials

From the Wheat Genetic & Genomic Resources Center (WGGRC) Kansas State University, USA , twenty-six *T. aestivum *Chinese Spring deletion lines were selected as described [22-24]. The deletion lines had partial deletions of the long and short arms of chromosomes 1 and 6, which was characterized by cytogenetics (Figures 2C and 4B). One line contained deletions of both the short arm of chromosome 1 and chromosome 6 (1BS-19/6DS-4, Figure 2C). All deletion lines were grown in containment glasshouses. No morphological differences were observed. Seeds were harvested from mature wheat plants.

### Database search for the specificity of the sequences recognized by mAbs compared to T-cell epitopes

The frequency of occurrence of known T-cell epitopes involved in the onset of CD was analyzed by searching within the National Center for Biotechnology Information (NCBI) database. From the NCBI protein database  five different groups of gluten protein sequences were extracted and subsequently converted into FASTA formats, using the following search queries: 'alpha gliadin', 'gamma gliadin', 'omega gliadin' 'D-type LMW-GS', 'LMW glutenin', and 'HMW glutenin'. All non-*Triticum*, non-*Aegilops *entries, and sequences containing less than 100 amino acids were removed. For the 'HMW glutenin' group only full size sequences were analyzed. The obtained protein sequences were aligned using ClustalW to validate if the correct groups were assigned to the sequences. Within the 'gamma gliadin' group, four sequences (AAA34286, P04729, P04730, and AAA34285) were more similar to LMW glutenins and were transferred to the 'LMW glutenin' group. In the 'omega gliadin/D-type LMW-GS' group, one sequence (ABI20696) was specific for the 'alpha gliadin' group and was transferred to the 'alpha gliadin' group. The sequences in the five established groups were analyzed for the different minimal recognition sequences of mAbs and T-cells [25]. No mismatches were allowed. Scores were expressed as the number of sequences and as the percentage of the sequences in the established group that contained one or more recognition sequences. The T-cell minimal recognition sequences used in the analyses were: Glia-α9 (PFPQPQLPY), Glia-α20 (FRPQQPYPQ), LMW-glt (PFSQQQQSPF), HMW-glt (QGYYPTSPQ) and mAb minimal recognition sequences used were: Glia-α9 (QPFPQPQ), Glia-α20 (RPQQPYP), LMW-1 (PPFSQQ), LMW-2 (QSPF), HMW-glt (QGQQGYYP) [25-27,60].

### Extraction of gluten proteins

Gluten proteins were extracted from wheat grains according to Van den Broeck et al. [61]. Grains were ground in an analytical mill (A 11 Basic, IKA-Werke) and sieved through mesh (0.5 mm). Gluten proteins were extracted from 50 mg wheat flour by addition of 0.5 ml of 50% (v/v) aqueous iso-propanol with continuous mixing (MS1 Minishaker, IKA Works, Inc.) at 1000 rpm for 30 min at room temperature, followed by centrifugation at 10,000 rpm for 10 min at room temperature. The residue was re-extracted twice with 50% (v/v) aqueous iso-propanol, 50mM Tris-HCl, pH 7.5 containing 1% (w/v) DTT, for 30 min at 60°C with mixing every 5 to 10 min followed by centrifugation at 10,000 rpm for 10 min at room temperature. After addition of each next extraction solution, the residue was resuspended by shaking in a Fastprep^® ^FP220A Instrument for 10 sec at 6.5 m/sec followed by sonication for 10 min in an ultrasonic bath (Branson 3510, Branson Ultrasonics Corporation). The three obtained supernatants were combined and considered the gluten protein extract. The protein content was quantified using the Biorad Protein Assay (Bio-Rad Laboratories), based on the Bradford dye-binding procedure, according to manufacturer's instruction with BSA as a standard.

### SDS-PAGE

Gluten proteins were separated on SDS-PAGE gels (10%) using a SE260 mighty small II system (GE Healthcare, UK). SDS-PAGE was followed by immunoblotting or by silver staining [62] with some modifications. Gels were fixed in 50% (v/v) ethanol/10% (v/v) acetic acid in water for 30 min. Then, gels were washed in 5% (v/v) ethanol/1% (v/v) acetic acid in water for 10 min, followed by three times washing for 5 min in MilliQ water. Gels were sensitized in 0.02% (w/v) sodium thiosulfate for 1 min and again washed three times for 30 sec in MilliQ water. Gels were incubated in 0.1% (w/v) silver nitrate for at least 20 min. After this incubation, gels were rinsed 2 times for 5 sec in MilliQ water and developed in 6% (w/v) sodium carbonate containing 0.05% (v/v) formaldehyde (37%)/0.4‰ (w/v) sodium thiosulfate. Development of staining was stopped by addition of 5% HAc/water.

### Immunoblotting

Proteins were blotted onto nitrocellulose (0.2 μm, Bio-Rad Laboratories), in buffer omitting methanol, using a Mini Trans-Blot Cell (Bio-Rad Laboratories) at 100 V for 1 hour. Blots were incubated and visualized as described [63] using mAbs specific for T-cell stimulatory epitopes against Glia-α9 [26,60], Glia-α20 [25,60], GLT-156 (LMW-1 and LMW-2) [27,60], HMW-glt [26,60]. Monoclonal Ab binding was visualized by staining for alkaline phosphatase, using Nitro Blue tetrazolium (NBT) and 5-Bromo-4-chloro-3-indolyl phosphate (BCIP) (Sigma).

### Quadrumat milling

To obtain white wheat flour, wheat kernels (total weight ranging 7.6–36 g) were milled using a Quadrumat JR (Brabender, Germany). Kernel moisture was adjusted to 16.5%. Bran was separated from endosperm flour by sieving through mesh (150 μm). After sieving the average yield was 50% (w/w), noting that samples 6AS-1 and 6DS-2 had a typically higher flour yield of 64% and 60%, the other samples ranged from 43% to 51%.

### Total protein content in flour

Flour protein content was estimated by the Dumas method [64] using an NA2100 Nitrogen and Protein Analyzer (ThermoQuest-CE Instruments, Rodeno, Italy). The Dumas method is based on the measurement of total nitrogen in the sample (N × 5.7). Methionine was used as a standard.

### Isolation of glutenin macro polymer from flour and glutenin particle size analysis

Dough strength is correlated to the amount of the *g*lutenin macro polymer (GMP) and to the size of glutenin particles. Glutenin macro polymer was isolated by dispersing wheat flour in 1.5% (w/v) SDS followed by ultracentrifugation as described [29]. Fresh GMP from flour was dispersed in 1.5% (w/v) SDS (10 ml) by rotating overnight at room temperature. Particle size distributions were measured using a Mastersizer 2000 (Malvern Instruments, UK). The laser diffraction pattern obtained with the instrument was correlated to the particle size distribution based on Fraunhofer theory, assuming a spherical particle shape. The range of the instrument was 0.02–2000 μm. Dispersions of GMP were transferred to the water filled sample vessel at an obscuration of approximately 8%. The surface area mean (D_3,2_) was used from the particle size distribution data for comparisons. Further details of this method are described by Don et al. and Wang et al. [31,65].

### Mixing experiments

Dough strength was determined using a micro-Mixograph. A 2 g Mixograph (National Manufacturing Co., USA) pin-mixer was used to analyze the mixing properties of the different flour samples. Mixing was performed at 20°C. Water was added according to the Plastograph method (ICC 115/1 (ICC, 1992) [66]. Dough contained 2% (w/w) sodium chloride (Merck, Germany). Bandwidth at peak resistance (BWPR) in percentages and dough development time (DDT) in minutes were used from the midline analysis for comparison.

### Flow-relaxation measurements

Relaxation tests were performed to study dough elasticity. Longer relaxation half times indicate more elastic dough behavior [32,33]. Dough was mixed to peak in the 2 g Mixograph pin-mixer, carefully removed from the mixer and transferred to the Bohlin VOR rheometer (Bohlin Instruments, Sweden). Flow-relaxation measurements were performed using an aluminum grooved plate geometry with a cross-section of 30 mm and a gap of 1 mm [33]. Moisture loss from the dough piece was prevented using paraffin oil. The actual measurement was performed after an equilibration time of 30 min to allow appropriate release of dough stress. The measuring temperature was 20°C. During measurement, the sample was deformed to a strain of 100% at a shear rate of 0.0208 s^-1^. The strain was kept constant and the subsequent decrease of stress of the dough was recorded as a function of time. The time necessary for the dough to relax to a stress of 50% of the initial stress, recorded directly after stopping deformation, was used as the flow-relaxation half time (T_1/2_).

## Authors' contributions

IMM and MJMS initiated this study. MJMS and CS selected Chinese Spring deletion lines. CS and HCB extracted gluten proteins from deletion lines. EMJS and TWJMH performed data base search. LD raised monoclonal antibodies. HCB performed SDS-PAGE and immunoblotting. TWJMH performed technological tests. IMM supervised HCB and CS. RJH, DB, MJMS, and LJWJG supervised TWJMH. HCB, TJWMH, RJH, MJMS, LJWJG, and IMM contributed to writing the manuscript. EMJS, LD, and DB gave editorial comments. All authors read and approved the final manuscript.

## Supplementary Material

Additional file 1 **Rheological parameters**. Rheological parameters of Chinese Spring wild type and deletion lines. Click here for file
