# Supplementary material for: Removing celiac disease-related gluten proteins from bread wheat while retaining technological properties: a study with Chinese Spring deletion lines
Source: BMC Plant Biol. 2009 Apr 7;9:41. doi: 10.1186/1471-2229-9-41 (PMC2670835; doi:10.1186/1471-2229-9-41)
Supplement: Additional file 1 — Rheological parameters. Rheological parameters of Chinese Spring wild type and deletion lines. [file 1471-2229-9-41-S1.doc]

Rheological parameters of Chinese Spring wild type and deletion lines.

|  | Total protein in flour (%) | DDT^a^ (min) | BWPR^b^ (%) | T½^c^ (sec) | GMP^d^ volume (µl/mg) | Particle surface area (D_3,2_) (µm) |
| --- | --- | --- | --- | --- | --- | --- |
| CS wild type | 11.7 (±0.2) | 3.1 (±0.3) | 18.3 (±0.5) | 45.0 (±3.0) | 10.2 (±0.2) | 12.2 (±0.2) |
| 1AL-1 | 14.7 (±0.4) | 3.6 (±0.2) | 27.3 (±0.2) | 48.7 (±0.2) | 12.2 (±0.5) | 15.9 (±0.3) |
| 1BL-1 | 12.9 (±0.7) | 2.1 (±0.1) | 19.1 (±0.8) | 30.9 (±0.5) | 1.4 (±0.2) | NA |
| 1DL-4 | 17.0 (±1.0) | 1.6 (±0.1) | 17.6 (±0.0) | 17.5 | 0.3 (±0.1) | NA |
| 1AS-1 | 15.2 (±0.5) | 4.0 (±0.4) | 23.0 (±2.0) | 67.2 (±0.5) | 11.7 (±0.0) | 11.9 (±0.1) |
| 1BS-10 | 13.0 (±2.0) | 2.8 (±0.2) | 22.4 (±0.6) | 42.2 (±0.5) | 7.3 (±0.1) | 11.2 (±0.2) |
| 1DS-1 | 13.0 (±2.0) | 3.0 (±0.1) | 24.9 (±0.1) | 45.8 (±0.3) | 12.6 (±0.0) | 12.4 (±0.2) |
| 6AS-1 | 20.5 (±0.8) | 3.0 (±0.0) | 42.9 (±0.9) | 14.7 | 12.0 (±1.0) | 18.2 (±0.1) |
| 6BS-1 | 14.3 (±0.4) | 4.7 (±0.1) | 23.2 (±0.2) | 36.8 (±0.5) | 10.8 (±0.1) | 10.9 (±0.1) |
| 6DS-2 | 14.2 (±0.8) | 4.1 (±0.3) | 27.3 (±0.7) | 33.9 | 8.7 (±0.7) | 10.5 (±0.1) |

All technological measurements were performed in duplicate, except the relaxation test (T½) for deletion lines 1DL-4, 6AS-1, 6DS-2 and 6DS-4/1BS-19. Errors represent the standard errors. NA in cell means not analyzed.

^a^Dough development time

^b^Band Width at Peak Resistance

^c^Flow-relaxation half time

^d^Glutenin Macro Polymer
